# Synthesis of 3,4-Dihydropyrimidin-2(1H)-Ones and Their Corresponding 2(1H)Thiones Using Trichloroacetic Acid as a Catalyst under Solvent-Free Conditions

**DOI:** 10.5402/2012/474626

**Published:** 2012-10-15

**Authors:** Zahed Karimi-Jaberi, Mohammad Sadegh Moaddeli

**Affiliations:** Department of Chemistry, Firoozabad Branch, Islamic Azad University, P.O. Box 74715-117, Firoozabad, Fars, Iran

## Abstract

Trichloroacetic acid was found to be a convenient catalyst for the synthesis of 3,4-dihydropyrimidin-2-(1H)-ones and their corresponding 2(1H)-thiones through a one-pot three-component reaction of aldehydes, alkyl acetoacetate, and urea or thiourea at 70°C under solvent-free conditions.

## 1. Introduction

Biginelli reaction is a useful three-component reaction offering versatile protocol for the production of 3,4-dihydropyrimidin-2(1H)-ones which exhibit widespread biological applications such as antihypertensive, antiviral, antitumor, antibacterial, *α*-1a-antagonism, antioxidant, and anti-inflammatory actions [9, 10].

Although numerous catalysts have been developed in accelerating this reaction [1–8, 11–22], it is still desirable to develop this reaction using newer reagents with greater efficiency, simpler operational procedure, and milder reaction condition, and a higher yield of products coupled with potential bioactivity is important.

**Scheme 1:**
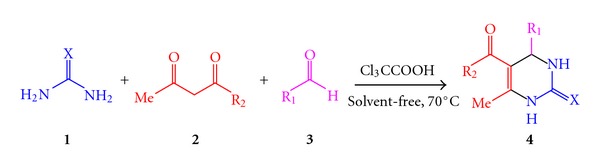


With the awareness of environmental issues and importance of this reaction and keeping our interest in the development of synthetic routes to heterocyclic compounds [23–27], herein, we report a heterogeneous, solid trichloroacetic acid, as an alternative, cheap, and efficient catalyst for the Biginelli reaction (Scheme 1).

Trichloroacetic acid is a readily available and inexpensive solid reagent and it has been used by our group for the synthesis of dihydropyrano[2,3-c]pyrazoles [23] and tetrahydrobenzo[a]xanthen-11-ones and dibenzo[a,j]xanthenes [24].

## 2. Results and Discussion 

The catalytic activity of trichloroacetic acid was first investigated using three-component reaction of benzaldehyde, ethyl acetoacetate, and urea as a model reaction. After carrying out the reaction at different conditions, the best results have been obtained with 20 mol% trichloroacetic acid at 70°C after 4 min with 85% yield under solvent-free conditions. In the absence of trichloroacetic acid, only 20% yield of the product was obtained even after heating at 70°C for 12 h with recovery of starting material.

The reaction was also examined in solvents such as EtOH, H_2_O, CHCl_3,_ and toluene. In the presence of solvents, reaction was sluggish and the formation of by-products was observed. The reaction temperature was also optimized, below 70°C the reaction proceeded slow giving a relatively low yield and no improvement was observed above 70°C.

Having established the reaction conditions, various 3,4-dihydropyrimidin-2(1H)-ones were synthesized in excellent yields through the reaction of different aldehydes, alkyl acetoacetate, and urea. The results are summarized in Table 1, which clearly indicates the generality and scope of the reaction with respect to various aromatic, heteroaromatic, unsaturated, and aliphatic aldehydes. It is noteworthy that acid-sensitive aldehydes such as furfural and cinnamaldehyde that (Table 1) worked well gave the corresponding products. The reaction can also proceed with methyl acetoacetate (Table 1, entries 18–30). In all cases, dihydropyrimidinones were the sole products and no by-product was observed.

The reaction of aldehydes with alkyl acetoacetate and thiourea under similar reaction conditions also provided the corresponding 3,4-dihydropyrimidin-2(1H)-thiones in high yields (Table 1, entries 31–35), which are also of interest with respect to their biological activities [21].

## 3. Conclusion

In conclusion, a novel approach to explore the use of trichloroacetic acid for the synthesis of 3,4-dihydropyrimidin-2-(1H)-ones and their corresponding 2(1H)thione has been described through the Biginelli reaction at 70°C under solvent-free conditions. This method offers several advantages including high yields, short reaction times, solvent-free condition, a simple work-up procedure without using any chromatographic methods, and it also has the ability to tolerate a wide variety of substitutions in all three components.

## 4. Experimental

All chemicals were commercially available and used without further purification. Melting points were recorded on an electrothermal type 9100 melting point apparatus. The IR spectra were obtained on a 4300 Shimadzu spectrophotometer as KBr disks. The NMR spectra were recorded on a Bruker 250 MHz spectrometer.

### 4.1. General Procedure for the Synthesis of 3,4-Dihydropyrimidin-2(1H)-Ones/Thiones

A mixture of aldehyde (1 mmol), alkyl acetoacetate (1 mmol), urea/thiourea (1 mmol), and trichloroacetic acid (0.032 g, 20 mol%) was stirred at 70°C for the appropriate time indicated in Table 1. The progress of reactions was monitored by TLC (ethyl acetate/n-hexane). After completion of the reaction, a solid was obtained. It was allowed to cool to room temperature, and ethanol (5 mL) was added, and the catalyst was recovered by filtration. The filtrate was concentrated and allowed to crystallize the desired product.

### 4.2. Selected Characterization Data

**Ethyl-6-Methyl-2-Oxo-4-Phenyl-1,2,3,4-Tetrahydropyrimidine-5-Carboxylate**

IR(KBr):3240, 3110, 1725, 1700, 1645; ^1^H NMR (DMSO-*d*6): *δ* 1.12 (t, *J* = 7.5 Hz, 3H), 2.28 (s, 3H), 4.03 (q, *J* = 7.5 Hz, 2H), 5.17 (d, *J* = 3.0 Hz, 1H), 7.22–7.41 (m, 5H), 7.78 (br s, 1H), 9.22 (br s, 1H).

**Ethyl-6-Methyl-4-(4-Nitrophenyl)-2-Oxo-1,2,3,4-Tetrahydropyrimidine-5-Carboxylate**

IR(KBr):3230, 3120, 1730, 1710, 1650; ^1^H NMR (DMSO-*d*6): *δ* 1.11 (t, *J* = 7.5 Hz, 3H), 2.29 (s, 3H), 4.00 (q, *J* = 7.5 Hz, 2H), 5.29 (d, *J* = 3.0 Hz, 1H), 7.51 (d, *J* = 10 Hz, 2 H), 7.91 (br s, 1H), 8.23 (d, *J* = 10.0 Hz, 2H), 9.37 (br s, 1H).

**Ethyl-6-Methyl-4-(4-Methoxyphenyl)-3,4-Dihydropyrimidin-2(1H)-One-5-Carboxylate**

IR (KBr): 3390, 3243, 3106, 2958, 1706, 1651,1278, 1088. ^1^H NMR (DMSO-*d*6): *δ* 1.01–1.20 (t, 3H, *J* = 7 Hz, CH_2_CH_3_), 2.30 (s, 3H, CH_3_), 3.80 (s, 3H, OCH_3_), 3.90–4.20 (q, 2H, *J* = 7 Hz, CH_2_CH_3_), 5.60 (s, 1H, C4-H), 6.80–6.90 (d,2H, *J* = 7.2 Hz, ArH), 7.15–7.25 (d, 2H, *J* = 7.2 Hz, ArH), 7.65 (bs, 1H, NH), 9.17 (bs, 1H, NH).

**6-Methyl-4-Phenyl-3, 4-Dihydropyrimidin-2(1H)-Thione-5-Carboxylate**

IR (KBr): 3412, 3312, 3174, 3096, 2967, 1667, 1610, 1575. ^1^H NMR (DMSO-*d*6): *δ* 1.02–1.18 (t, 3H, *J* = 7.1 Hz, CH_2_CH_3_), 2.32 (s, 3H, CH_3_), 4.02–4.21 (q, 2H, *J* = 7.1 Hz,CH_2_CH_3_), 5.50 (s, 1H, C4-H), 7.15–7.35 (m, 5H, ArH), 8.90 (bs, 1H, NH), 9.95 (bs, 1H, NH).

## Figures and Tables

**Table 1:** Trichloroacetic acid catalyzed one-pot synthesis of 3,4-dihydropyrimidin-2-(1H)-ones or thiones under solvent-free conditions.

Entry	R_1_	R_2_	X	Time (min)	Yield (%)	mp (°C, obsd)	mp (°C, lit) (ref.)
1	C_6_H_5_	OEt	O	4	85	201–203	202–205 [1]
2	4-ClC_6_H_4_	OEt	O	9	92	212–216	210–212 [1]
3	4-HOC_6_H_4_	OEt	O	40	90	226–228	231–233 [2]
4	3-O_2_NC_6_H_4_	OEt	O	20	93	225–228	227-228 [1]
5	4-O_2_NC_6_H_4_	OEt	O	5	85	206–209	207–209 [1]
6	C_6_H_5_CH=CH	OEt	O	3	90	225–227	223–226 [1]
7	4-MeOC_6_H_4_	OEt	O	20	95	200–202	202–204 [1]
8	2,4-(Cl)_2_C_6_H_3_	OEt	O	4	91	247–249	246–248 [3]
9	4-MeC_6_H_4_	OEt	O	5	90	213–215	214–216 [1]
10	2-MeOC_6_H_4_	OEt	O	2	94	262-263	260 [4]
11	2,6-(Cl)_2_C_6_H_3_	OEt	O	3	96	226–228	226 [5]
12	2-ClC_6_H_4_	OEt	O	9	85	221–223	221–223 [2]
13	4-BrC_6_H_4_	OEt	O	11	90	212–214	215 [6]
14	CH_3_	OEt	O	3	92	188–190	194-195 [7]
15	CH_3_CH_2_CH	OEt	O	50	88	163–165	164–166 [7]
16	3-MeC_6_H_4_	OEt	O	8	93	219–222	224–226 [2]
17	2-Furyl	OEt	O	19	86	202–205	202–204 [5]
18	C_6_H_5_	OMe	O	5	94	208–211	210–213 [1]
19	4-MeOC_6_H_4_	OMe	O	9	85	192–195	193–196 [3]
20	4-ClC_6_H_4_	OMe	O	8	92	204–206	203–205 [1]
21	4-O_2_NC_6_H_4_	OMe	O	3	95	235–237	235-236 [5]
22	2-ClC_6_H_4_	OMe	O	6	84	180–182	181–183 [1]
23	3-O_2_NC_6_H_4_	OMe	O	12	90	271–274	273–275 [2]
24	4-MeC_6_H_4_	OMe	O	14	93	206–209	210–213 [3]
25	4-HOC_6_H_4_	OMe	O	7	87	235–237	231–233 [2]
26	2-MeOC_6_H_4_	OMe	O	2	95	284–286	285–287 [2]
27	3-MeC_6_H_4_	OMe	O	4	96	214–217	216–218 [2]
28	3-ClC_6_H_4_	OMe	O	9	92	208–211	209-210 [2]
29	2,4-(Cl)_2_C_6_H_3_	OMe	O	3	94	252–255	252-253 [3]
30	2-Furyl	OMe	O	11	88	216–218	214–216 [8]
31	C_6_H_5_	OEt	S	25	90	210–212	210–212 [1]
32	4-ClC_6_H_4_	OEt	S	18	86	181–183	184-185 [3]
33	4-MeOC_6_H_4_	OEt	S	20	85	136–138	137–139 [3]
34	3-O_2_NC_6_H_4_	OEt	S	15	87	205–208	205-206 [8]
35	C_6_H_5_	OMe	S	13	92	220–222	221-222 [3]
